# An Impeached Committee

**Published:** 1890-03-15

**Authors:** 


### The Hospital

A Weekly Journal of

### Science, Medicine, Nursing, and Philanthropy.

*For Hospitals, Asylums, Medical Practitioners, Students, Nurses, Families, Parishes, Congregations, and the Charitable Public.*

Vol. VII.—No. 181.] [March 15, 1890.

### An Impeached Committee.

The Earl of Chichester is the President of the Sussex County Hospital at Brighton; but it is not probable that his Lordship will be sent to the Tower, even though the impeachment of himself and his fellow-members of Committee by a "pamphleteer" governor of the Hospital should prove as successful as the latter seems to think it ought to prove. But though there are no very definite penalties attached to the proved misuse of public funds by Hospital Managers, yet such misuse is always discreditable, and, if carried to considerable lengths, is disgraceful. The Brighton Committee will, no doubt, take this view of the matter, and will be anxious, if possible, to disprove the charges made against them. If that be not possible, they will, as right-minded Englishmen, take the criticisms to heart, and devote more care and energy to the important work of managing properly a large County Hospital.

The governor, whose published pamphlet sets forth in detail the charges made against the Committee of Management, must expect the public to criticise his charges as freely as he has criticised the Hospital Managers. We shall give those charges in some detail, and then make such comments thereupon as our knowledge of facts seems to warrant. The writer, by way of enabling his readers to see the faults in the management of the Brighton Hospital, contrasts it with the administration of the Consumption Hospital at Brompton. For a full description of the management at Brompton, he refers his readers to the last Hospital Sunday Number of this journal. He practically affirms that just as the management at Brompton reaches the maximum of excellence, so does the mismanagement at Brighton sink to the lowest depths of demerit. He clinches this part of his argument by the cate gorical assertion that the cost per bed per annum at the Brighton Hospital exceeds that of any other similar institution by £14. He claims as his authority for this last statement the *Hospital Annual* for 1889.

Proceeding next to consider the question of the comfort of the patients, the accuser once more contrasts the state of things at Brompton with that at Brighton. At the former place the patients wait in a well-warmed room, where tea, coffee, and other refreshments can be had at merely nominal prices, and where they are made as comfortable as they can possibly be until their long and weary period of waiting is over. At Brighton, says the writer, "no such reception is accorded, and although many patients come from long distances they cannot obtain sup or bite within a quarter of a mile of the hospital; and if any go in quest of refreshment he or she may miss their turn, and consequently have long to wait." Some patients, it is said, have to sit for four hours before their turns arrive; and all that time, though sick and ill, they must endure the pangs of unrelieved hunger and thirst. The unfeeling conduct of the committee is brought out into sharp relief by the alleged fact that some residents at Brighton, pitying the distresses of the waiting patients—often women and children—recently offered to present to the hospital all the appliances necessary for a refreshment stall, provided sufficient space was given on which to erect it. "After weeks and months of waiting . . . the offer was declined by a resolution of the committee passed in June." Other charges of a minor kind are made, and then the writer proceeds to complain that no entertainments, such as concerts, dramatic recitals, penny readings, and the like, are even so much as allowed at the Brighton Hospital, although, as is well known, such entertainments are almost universal elsewhere, and are looked upon by medical men generally as among the most effective "aids to cure" known to their art. Of still deeper gravity than this is the charge that the corridors, offices, and bath-rooms throughout the hospital are never warmed, whilst even the wards themselves are for the most part provided only with old-fashioned fire-places, totally inadequate to the maintaining of a healthy temperature. The chapel, where all religious services are held, is declared to be a mere "ice house," in which even robust persons cannot sit through the service without the risk of contracting severe and dangerous colds.

To make the indictment complete the writer of the pamphlet alleges that not only is the money of the hospital wasted, but that subscribers and their subscriptions are alike neglected and despised. Little or no trouble is taken to collect the subscriptions of old subscribers, whilst the idea of making new ones seems never to have entered the minds of the committee at all. Many new subscribers, it is believed, might be had merely for the asking; many more, it is affirmed, would willingly respond to the claims of the institution if they had faith in the capacity of its managers. No charge is brought against the physicians and surgeons. On the contrary, it is asserted that the medical staff consists of "as skilful a body of men as can be found attached to any hospital in Europe," whilst the nurses and other officials are declared to be equally devoted and excellent.

Taken separately, any one of these charges would demand the attention of the committee of management of the Hospital; taken together, they constitute, if true, a public scandal. To the committee they are absolutely disgraceful, if true; and now that they are known, the disgrace will fall upon Brighton itself if its responsible inhabitants do not thoroughly investigate the questions raised.

But are these charges true? Let us take the first, which states, on the authority of the *Hospital Annual* that the cost per bed per annum at the Brighton Hospital exceeds that of any other institution by £14. That statement the *Hospital Annual* does not confirm. On the contrary, if the London general hospitals be taken for comparison, some of them show nearly double the cost per bed per annum of the Brighton Hospital. But the proper institutions to compare with that of Brighton are the provincial hospitals, and more especially those in country towns of similar size and character. Of those there are several that spend nearly as much per bed per annum as the Brighton Hospital, whilst some spend considerably more. Among these last may be mentioned the Macclesfield Infirmary, the Blackburn and East Lancashire Infirmary, and the Royal Portsmouth Hospital and Dispensary. But whilst this is pointed out for the sake of accuracy, it is fair to say that the Brighton Hospital exceeds the average expenditure per bed per annum of similar hospitals taken in the mass. At fifty provincial hospitals resembling in size and character that at Brighton, the average cost per head per annum, according to the *Hospital Annual* is £57 0s. 8d. The cost at Brighton is £71 14s. 8d. That, however, is very different from the statement that the cost per bed at Brighton exceeds the cost per bed at any other institution in the kingdom by £14.

With every desire to do justice to a writer who has taken so much interest in the Hospital, of which he is a governor, as to inquire into every detail of its management, to compare that management with the administration of other hospitals, and to publish the results of his investigations with the object of bringing about needed reforms, we cannot but feel that he has seriously weakened his case by the gravest possible inaccuracy in the leading count of his indictment. So far from proving that the Brighton Hospital is the most extravagant institution of its kind in the kingdom, he does not succeed in showing that it can properly be called extravagant at all. Of the accuracy of his other charges we, not being on the spot, have no means of forming a correct judgment. But if a winnowing process applied to them should produce the same result as has been produced by the winnowing of his main charge, it is evident that a very different aspect will be put upon the case from that which he has striven to depict. It is probable that so far as the want of a comfortable waiting-room and of a refreshment stall are concerned, the managers cannot be acquitted of blame. It seems clear, also, that the cold chapel, the unwarmed corridors, and the heating arrangements generally, demand the attention of the committee. If this be so, we would venture to plead on behalf of the poor patients that immediate steps be taken to prevent unnecessary distress, and probable injury to health. But to the writer of the pamphlet we cannot forbear repeating our often-expressed conviction that exaggerated charges which investigation will not support, do infinite mischief to noble institutions, and generally fail to accomplish the very objects the accusers have in view. Truth, plain truth, is what everybody owes, first to himself, and then to all the world.

